# Association between the *TOX3* rs3803662 C>T polymorphism and recurrent miscarriage in a southern Chinese population

**DOI:** 10.1002/jcla.22992

**Published:** 2019-08-27

**Authors:** Wendong Huang, Huazhong Zhou, Qisen Li, Lei Pi, Yufen Xu, LanYan Fu, Yanfang Yang, Zhaoliang Lu, Di Che, Xiaoqiong Gu

**Affiliations:** ^1^ Research Center Maoming People's Hospital Guangdong China; ^2^ Department of Clinical Biological Resource Bank, Guangzhou Institute of Pediatrics, Guangzhou Women and Children’s Medical Center Guangzhou Medical University Guangzhou China; ^3^ Department of Prenatal Diagnosis Maoming People’s Hospital Maoming Guangdong China; ^4^ Department of Clinical Lab, Guangzhou Women and Children’s Medical Center Guangzhou Medical University Guangzhou China; ^5^ Department of Blood Transfusion, Guangzhou Women and Children’s Medical Center Guangzhou Medical University Guangzhou China

**Keywords:** genetic susceptibility, recurrent miscarriage, rs3803662, *TOX3* gene

## Abstract

**Background:**

Studies have shown that some genetic polymorphisms associated with breast cancer susceptibility may also be associated with abortion. The *TOX3* gene plays a key role during the onset of breast cancer, and reproductive factors such as abortion are risk factors for breast cancer. However, there is currently no study describing the relationship between the *TOX3* rs3803662 C>T polymorphism and the risk of recurrent miscarriage. Therefore, we investigated whether the *TOX3* rs3803662 C>T polymorphism is associated with recurrent miscarriage susceptibility in this case‐control study.

**Methods:**

We recruited 248 recurrent miscarriage patients and 392 healthy controls from the southern Chinese population and performed genotyping using the TaqMan method.

**Results:**

The results showed no evidence that *TOX3* rs3803662 C>T is associated with recurrent miscarriage (CT and CC: corrected OR = 1.038, 95% CI = 0.737‐1.461, *P* = .8321; TT and CC: adjusted OR = 0.989, 95% CI = 0.591‐1.656, *P* = .9659; dominant model: adjusted OR = 1.027, 95% CI = 0.742‐1.423, *P* = .8712; recessive model: adjusted OR = 0.969, 95% CI = 0.600‐1.566, *P* = .8975).

**Conclusion:**

According to this study, the *TOX3* rs3803662 C>T polymorphism may not be associated with recurrent miscarriage in the southern Chinese population. A larger multicenter study is needed to confirm the results.

## 1 INTRODUCTION

Recurrent miscarriage (RM) is defined as two or more consecutive spontaneous abortions before the 20th week of gestation in women with the same sexual partner.^1^, ^2^ Although the cause of RM is still unclear, an increasing number of studies have shown that genetic susceptibility plays an important role.^3^, ^4^, ^5^ Therefore, exploration of the causes of recurrent abortion has important implications for the prevention and treatment of recurrent abortion. Recent studies have shown a link between abortion and breast cancer risk, and induced abortion may be a risk factor for breast cancer.^6^, ^7^ Moreover, there are studies confirming that breast cancer may be associated with reproductive risk factors.^8^ In addition, other studies have shown that certain genetic polymorphisms are associated with breast cancer and may also be related to abortion, for example, the *MDM2* Del1518 polymorphism and *miR‐196a* (rs11614913).^9^, ^10^, ^11^ Therefore, it is helpful to understand the causes of RM by studying whether some genes involved in breast cancer pathogenesis are associated with RM.

Located on 16q12.1, the *TOX3* gene is 143 kb long and consists of seven exons.^12^ Studies have shown that the genetic variants in the *TOX3* gene are associated with multiple diseases, including lung cancer, familial ovarian cancer, polycystic ovary syndrome (PCOS), and breast cancer,^13^, ^14^, ^15^, ^16^, ^17^, ^18^, ^19^ and Wang Q et al found that the rs3803662 C>T polymorphism is strongly related to an increased risk of breast cancer in both Asian and Caucasian populations.^20^ Moreover, studies have confirmed that TOX3 methylation abnormalities may be closely related to the occurrence of PCOS and may play a role in the development of PCOS pathology by regulating changes in TOX3 protein expression.^21^ In recent years, studies have confirmed that rs3803662 C>T is the most important genetic variation in the *TOX3* locus, which is associated with an increased risk of breast cancer in women.^20^, ^22^ The SNP rs3803662 C>T also shows significant associations with estrogen receptor status,^23^ and patients with PCOS have a higher risk of spontaneous abortion.^24^ Additionally, reproductive factors such as abortion are known risk factors for breast cancer.^25^, ^26^ A recent study of ours found that some lncRNA gene polymorphisms associated with breast cancer susceptibility are also associated with RM, such as *lncRNA CCAT2* and *lncRNA MALAT1*.^27^, ^28^ However, it remains unclear whether the TOX3 gene is also associated with recurrent abortion susceptibility, similar to lncRNA CCAT2. These previous studies suggest that genetic variation in the *TOX3* locus may be associated with susceptibility to RM. However, there is currently no study on the relationship between the *TOX3* rs3803662 C>T polymorphism and the risk of RM. These findings prompted us to evaluate whether the rs3803622 variant of the *TOX3* gene is associated with RM susceptibility and whether rs3803622 C>T can be used as a biomedical indicator or a potential risk factor.

## 2 MATERIALS AND METHODS

### 2.1 Study subjects

This study included 248 women diagnosed with RM at the Guangzhou Women and Children's Medical Center between June 2017 and July 2018. Diagnostic criteria were defined as two or more consecutive pregnancy losses before 20 weeks of gestation.^29^ A total of 392 age‐matched healthy controls who had at least two normal pregnancies and did not have a history of miscarriage were recruited from the Gynecology Department of Guangzhou Women and Children's Medical Center between June 2017 and July 2018. Women with a history of autoimmune disease; liver and kidney dysfunction; metabolic disorders; endocrine, uterine anomalies, arterial, or venous thrombosis; and other diseases were excluded from the healthy control and RM groups.

### 2.2 Genotyping and DNA extraction

All blood samples were supplied by the Clinical Biological Resource Bank of the Guangzhou Women's and Children's Medical Center. Total genomic DNA was extracted from blood using a TIANamp Blood DNA Kit (Tiangen, Beijing, China). An ABI Q6 instrument (QuantStudio™ 6 Flex Real‐Time PCR System, Applied Biosystems) and TaqMan assays were used to genotype the rs3803622 polymorphism according to the real‐time polymerase chain reaction protocol. The typing probe rs3803622 was purchased from Applied Biosystems (Applied Biosystems TaqMan). The ID number of rs3803622 is ID: C__25968567_10.

### 2.3 Statistical analysis

Hardy‐Weinberg equilibrium (HWE) in the control group was confirmed by the goodness‐of‐fit chi‐square test. Unconditional univariate logistic regression analysis was applied to analyze genotypic and demographic differences between the RM patients and healthy individuals. Furthermore, the association between rs3803622 C>T polymorphism and susceptibility to RM was assessed by the 95% confidence interval (CI) and odds ratio (OR). A stratified analysis of age and number of abortions was performed, and all statistical tests were bilateral. SAS software (version 9.4; SAS Institute) was employed for all statistical analyses (*P* values <.05 were considered statistically significant).

### 2.4 Ethics statement

The study was approved by the Medical Ethics Committee of Guangzhou Women and Children's Medical Center. Written informed consent was obtained from each RM patient and control subject before participation in the study.

## 3 RESULTS

### 3.1 Demographic characteristics

The demographic characteristics of the RM patients (approximately 68.15% of RM patients experienced two or three spontaneous abortions, and more than 31.85% experienced four or more) and healthy controls are listed in Table 1. Among the 248 RM patients and 392 healthy controls included, no significant differences in age were found (31.00 ± 4.83 vs 31.44 ± 4.39 years, *P* = .7225).

**Table 1:** Frequency distribution of selected characteristics in recurrent miscarriage and control groups

Variables	Cases (n = 248)		Controls (n = 392)		*P* a
	No.	%	No.	%	
Age range, y	20‐44		22‐44		
Mean ± SD	31.00 ± 4.83		31.44 ± 4.39		.7225
**<**35	187	75.4	288	73.47	
35‐40	52	20.97	92	23.47	
>40	9	3.63	12	3.06	
No. of abortion/%					
2‐3	169	68.15			
≥4	79	31.85			

a Two‐sided chi‐square test for distributions between recurrent miscarriage patients and controls.

### 3.2 Association between TOX3 polymorphisms and RM susceptibility

The genotype distribution of the *TOX3* rs3803662 C>T polymorphism in RM patients and controls is shown in Table 2. The control group showed HWE for the *TOX3* rs3803662 C>T genotypes (HWE = 0.636). However, there was no significant association between the *TOX3* rs3803662 C>T polymorphism and age‐adjusted RM susceptibility (CT and CC: corrected OR = 1.038, 95% CI = 0.737‐1.461, *P* = .8321; TT and CC: adjusted OR = 0.989, 95% CI = 0.591‐1.656, *P* = .9659; dominant model: adjusted OR = 1.027, 95% CI = 0.742‐1.423, *P* = .8712; recessive model: adjusted OR = 0.969, 95% CI = 0.600‐1.566, *P* = .8975).

**Table 2:** Genotype and allele frequencies of TOX3 in RM patients and controls

Genotype/ allele	RM (N = 248）	Controls (N = 392)	*P‐*value^a^	OR (95% CI)	*P‐*value	Adjusted OR (95% CI)	*P‐*value^b^
TOX3/rs3803662 C>T (HWE = 636)							
CC	97 (39.11)	156 (39.80)		1.00	–	1.00	–
CT	120 (48.39)	186 (47.45)	–	1.038 (0.737‐1.461)	.8325	1.038 (0.737‐1.461)	.8321
TT	31(12.50)	50(12.76)	–	0.997 (0.596‐1.668)	.9913	0.989 (0.591‐1.656)	.9659
Additive			.9735	1.010 (0.796‐1.280)	.9373	1.007 (0.794‐1.276)	.9567
Dominant	151 (60.89)	236 (60.20)	.8633	1.029 (0.743‐1.425)	.8634	1.027 (0.742‐1.423)	.8712
Recessive	217 (87.50)	342 (87.24)	.9246	0.977 (0.605‐1.578)	.9248	0.969 (0.600‐1.566)	.8975

a Chi‐square test for genotype distributions between recurrent miscarriage patients and controls.

b Adjusted for age.

### 3.3 Stratification analysis

Stratified analysis in which the subjects were stratified by age and the number of abortions was conducted to further evaluate the effects of the *TOX3* rs3803662 C>T polymorphism in RM patients and controls (Table 3). The results showed that the *TOX3* rs3803662 C>T polymorphism was not significantly associated with RM risk in different age groups or based on the number of abortions.

**Table 3:** Stratification analysis for associations between TOX3 polymorphism and recurrent miscarriage risk in a south Chinese population

Variable	rs3803662 (cases/controls)		*P* a	OR (95% CI)	*P*	Adjust OR (95% CI)	*P* b
	CT/TT	CC					
Age							
<35	114/178	73/110	0.8537	0.965 (0.661‐1.408)	0.8535	–	–
35‐40	32/52	20/40	0.5568	1.231 (0.615‐2.464)	0.5578	–	–
>40	5/6	4/6	0.8007	1.250 (0.221‐7.084)	0.8009	–	–
No. of abortion/%							
2‐3	62/156	107/236	0.4872	1.141 (0.786‐1.656)	0.4883	1.135 (0.781‐1.650)	0.5074
4≥	35/156	44/236	0.4582	0.831 (0.510‐1.353)	0.457	0.836 (0.513‐1.362)	0.4719

a Chi‐square test for genotype distributions between recurrent miscarriage patients and controls.

b Adjusted for age.

## 4 DISCUSSION

Genetic susceptibility may be involved in the onset of recurrent miscarriage and play an important role in the occurrence and development of RM 30; such genes include *FOXP3, IL‐10,* and *TNF‐α*.^31^, ^32^, ^33^, ^34^ However, few studies have explored the association of long noncoding RNA (lncRNA) polymorphisms and RM. To the best of our knowledge, this study is the first to investigate the relationship between an lncRNA *TOX3* gene polymorphism (rs3803662 C>T) and susceptibility to this condition.

Although the function of the *TOX3 (TNRC9)* gene is unclear, many studies have found that the rs3803662 polymorphism is associated with multiple diseases. For example, a genome‐wide association study showed that the *TOX3* rs3803662 C>T polymorphism increases the risk of breast cancer in the Chinese Han population.^35^ And, Wang Q et al^20^ found that the rs3803662 C>T polymorphism is strongly related to an increased risk of breast cancer in both Asian and Caucasian populations. Moreover, Chen F et al^36^ reported that the T allele of the single‐nucleotide polymorphism (SNP) rs38033662 is significantly associated with an increased risk of breast cancer in Chinese Han women. Furthermore, Park SL et al^37^ found that *TOX3* rs3803662 C>T was significantly associated with the risk of breast cancer. Interestingly, Liao J et al^38^ did not observe a significant association between the rs3803662 C>T polymorphism and breast cancer in the southern Chinese population. These studies suggest that the relationship between the rs3803662 C>T polymorphism and breast cancer susceptibility has not yet been resolved. A recent study by Che et al^28^ showed that a genetic polymorphism of the *lncRNA CCAT2*, which is associated with breast cancer susceptibility, is also associated with recurrent miscarriage, and it is possible that similar to *lncRNA CCAT2,* the TOX3 gene is also associated with recurrent abortion susceptibility. Nonetheless, in our present case‐control study, we did not find any significant association between the rs3803662 polymorphism of the *TOX3* gene and susceptibility for recurrent abortion. This may be related to the different role of the rs3803662 C>T polymorphism in different diseases. Larger sample sizes and multicenter cohorts are needed for verification.

The study found that as the maternal age of pregnant women increases, the risk of miscarriage increases, and maternal age is an important risk factor for spontaneous abortion.^39^ At the same time, some studies have found that rs619586 AG/GG variants in the lncRNA *MALAT1* gene are more protective for women younger than 35 years old and women with 2‐3 miscarriages than rs619586 AA variants^26^. However, the present study showed that *TOX3* rs3803662 C>T was not associated with RM or with the number of prior miscarriages and age. Miscarriage is more prevalent in women older than 40 years,^39^, ^40^, ^41^ and women with previous induced abortion have an increased risk of miscarriage.^42^, ^43^ Our results indicate no difference in the rate of rs3803662 polymorphism in different age groups, and there were also no significant correlations between the rs3803662 C>T polymorphism and the number of prior abortions in stratified analysis. According to the results of this study, the rs3803662 polymorphism may not be a biomarker for miscarriage. Nonetheless, further studies with larger sample sizes are needed to confirm these results.

The current research has some limitations. First, the study was limited to the southern Chinese population and did not assess cases and controls among other ethnic groups. Given the large differences in the incidence of miscarriage in different races, the distribution and function of rs3803662 C>T in different populations need further study. Second, the analysis of miscarriages included age and the number of abortions, but due to lack of information, other factors such as family history were not considered in the stratified analysis, and such factors may affect the outcome of this study. Third, we only studied the relationship between rs3803622 C>T polymorphism in the *TOX3* gene and susceptibility to RM. The role of the *TOX3* gene in fetal implantation, fetal growth maintenance, or gestational immune balance was not investigated. Finally, the sample size in our current study is relatively small. A larger sample size is needed in future studies to confirm the role of rs3803622 C>T in the *TOX3* gene in susceptibility to recurrent abortion.

In summary, we conducted a case‐control study with a small sample size of 248 patients and 392 healthy controls from the southern Chinese population. The association between *TOX3* rs3803662 C>T polymorphism and miscarriages and other parameters, such as the number of abortions and the age at which miscarriages occurred, was not significant. A larger multicenter study is needed to confirm this conclusion.

## CONFLICT OF INTEREST

The authors report no conflicts of interest.

## AUTHOR CONTRIBUTIONS

All authors contributed significantly to this work. DC and WDH devised the research plan. The data were analyzed by YFX and YFY. DC wrote the study, and QSL, ZLL, and HZZ were responsible for performing the experiments. LP and LYF designed the experimental methods, and XQG modified and polished the study. All authors support the publication of the manuscript.
